# Ant Navigation: Fractional Use of the Home Vector

**DOI:** 10.1371/journal.pone.0050451

**Published:** 2012-11-29

**Authors:** Allen Cheung, Lex Hiby, Ajay Narendra

**Affiliations:** 1 Queensland Brain Institute, The University of Queensland, Brisbane, Queensland, Australia; 2 Conservation Research Ltd., Gt. Shelford, Cambridge, United Kingdom; 3 ARC Centre of Excellence in Vision Science, Research School of Biology, The Australian National University, Canberra, Australian Capital Territory, Australia; Lund University, Sweden

## Abstract

Home is a special location for many animals, offering shelter from the elements, protection from predation, and a common place for gathering of the same species. Not surprisingly, many species have evolved efficient, robust homing strategies, which are used as part of each and every foraging journey. A basic strategy used by most animals is to take the shortest possible route home by accruing the net distances and directions travelled during foraging, a strategy well known as path integration. This strategy is part of the navigation toolbox of ants occupying different landscapes. However, when there is a visual discrepancy between test and training conditions, the distance travelled by animals relying on the path integrator varies dramatically between species: from 90% of the home vector to an absolute distance of only 50 cm. We here ask what the theoretically optimal balance between PI-driven and landmark-driven navigation should be. In combination with well-established results from optimal search theory, we show analytically that this fractional use of the home vector is an optimal homing strategy under a variety of circumstances. Assuming there is a familiar route that an ant recognizes, theoretically optimal search should always begin at some fraction of the home vector, depending on the region of familiarity. These results are shown to be largely independent of the search algorithm used. Ant species from different habitats appear to have optimized their navigation strategy based on the availability and nature of navigational information content in their environment.

## Introduction

Path integration (PI) is a strategy used by many animals to return home by the shortest possible route. In path integration, animals compute a home vector (HV) by integrating the angles steered and distances travelled on the outward journey [1], [2], [3], [4]. The most conclusive evidence of an animal’s ability to path-integrate comes from experiments where individual animals returning home are displaced to a distant location where familiar visual landmark information is absent. If an animal continues to travel in the direction where the nest would have been it can be concluded that it has a path integrator. The path integrator accumulates both systematic and random errors and hence often leads animals to the vicinity of the home, rather than the home itself [5], [6], [7], [8]. It is perhaps to overcome such errors in the path integrator, animals rely on visual landmarks [9], [10] and use distinct search strategies [11], [12], [13], [14], [15], [16], [17], [18] to locate home.

**Figure 1:**
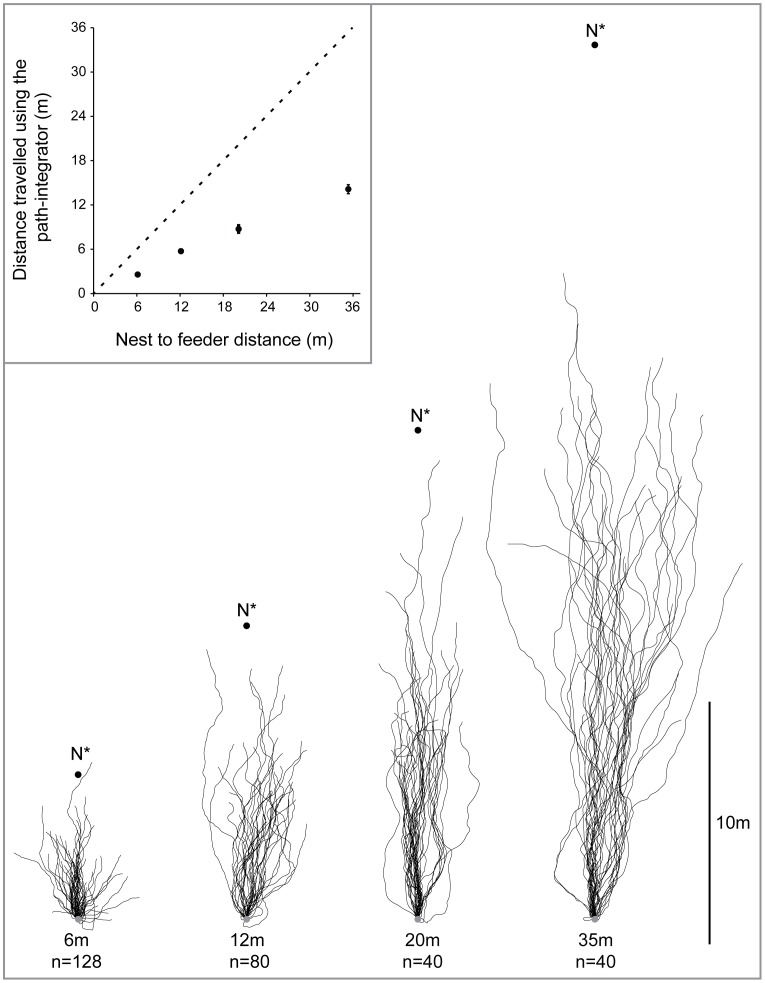
Distance travelled using the path integrator by *M. bagoti* ants. Homing trajectories of ants caught at feeder placed 6 m, 12 m, 20 m and 35 m from the nest. End point of the trajectories indicates the start of search. Fictive nest position (N*) and release point (R) is indicated. Inset: means±se of distance travelled before the start of search for each of the four distances from the nest to feeder. Dashed line indicates predicted path integration if animals had travelled the entire HV. Modified from Narendra 2007a [29].

Desert ants and most likely other ants too possess a path integration system. In landmark-poor habitats ants return home by taking the shortest possible route, thus relying on path integration (e.g., [5]). In landmark-rich habitats ants return home by establishing idiosyncratic paths using visual landmark information (for *Cataglyphis fortis* see [9], for *Melophorus bagoti* see [19]). Typically for a homing ant, both the path integrator and visual landmarks provide the same directional information. But when the two strategies are put in conflict, then either the path integration information is fully suppressed (e.g., [20], [21]), or ants follow a direction intermediate to that indicated by the path integrator and the visual landmarks (e.g., [21], [22], [23]). In principle, ants could find their way to the nest or back to the familiar route by moving to match the current view on their retina to a previously stored image either from a location along the route or from the nest (e.g., [24]). Such views can guide individual ants to return to the nest from long distances [25], [26]. When ants are displaced to distant locations where familiar visual landmarks are absent, their initial path is guided solely by the path integrator. During such distant displacements, the distance an ant travels following the home vector varies with the complexity of the landscape. For instance, in landmark-dense habitats of French Guiana ants travel only about 50 cm before beginning a search [27], [28], in semi-arid Central Australian deserts ants travel about 40% of their HV (Figure 1, [29]) and in landmark-poor habitats of North Africa ants travel nearly 90% of their HV [6]. The distance travelled by individual ants (*Melophorus bagoti*) relying on their HV differs even within the same species: fractional use of the HV increases from 40% in landmark-rich habitats to 70% in landmark-poor habitats [30]. Furthermore, when the outward and return journeys are restricted to homogeneous linear channels ants travel the entire distance of outbound path prior to initiating search [28], demonstrating the availability of a full HV.

Given these differences in the distance travelled relying on the HV, we here ask what the theoretically optimal balance between PI-driven and landmark-driven navigation should be. In the presence of a familiar route, we investigate the possibility that initiating search prior to running off the entire HV may be a robust solution to minimize the expected cost and maximize the probability of success in finding the familiar route.

**Figure 2:**
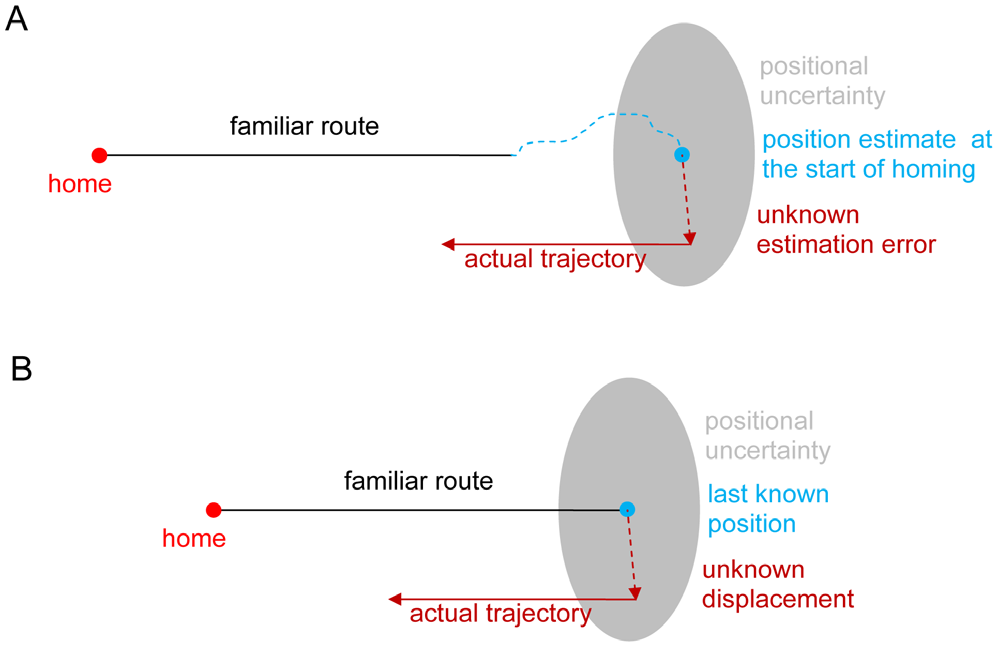
Positional uncertainty at the start of homing. A. Positional uncertainty due to errors accumulated during outbound foraging beyond the familiar route. B. Positional uncertainty due to sudden displacement from the end of the familiar route.

## Methods

Two models are used to determine the theoretically optimal point, according to the path integrator, at which to begin searching for home. In both models, it is assumed that.

- the navigating agent is familiar with an entire foraging route, i.e., if it is somewhere along a familiar route, it is able to find its way directly to its nest;
- the familiar route is approximately a straight line extending from its nest to some distance 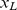;
- at some point prior to the homeward journey, the navigating agent is displaced from its familiar route (otherwise it would not need to search according to assumption (i));
- at the beginning of the homeward journey, the expected displacement is zero, i.e., over a large number of trials, the centre of the displacement distribution is unbiased.

The cause of displacement is not important to the theoretical modelling in this work but two scenarios are presented to motivate the need for an effective coupling between search and PI strategies for animal homing (Figure 2). There are at least two major types of mechanisms by which a navigating animal may be displaced from its familiar route. Firstly, the animal may have wandered away from the familiar route and can only maintain an erroneous estimate of its current position relative to its home (Figure 2A). Since it is now in an unfamiliar area, the animal’s navigation system accumulates uncertainty in position so that its best estimate of the HV is erroneous. Secondly, an animal could be displaced suddenly either by natural forces such as a wind gust, or by an experimentalist (Figure 2B). Assuming the PI system is unable to track the displacement, the best estimate of current position is the last known position, plus some unknown error. Both types of mechanisms result in a discrepancy between the true position of the animal, and its best estimate of current position. Since the animal’s navigation system only has access to the erroneous estimate of current position, the actual homing trajectory is displaced by the same discrepancy (or error) relative to the ideal homing trajectory.

Two models are used to determine the ideal HV distance to follow before initiating search, given that the true homing trajectory is displaced according to some error distribution (Figure 2, grey regions), which is unknown to the navigation system. The motivations and assumptions of the individual models are described below.

### 1. Cost Minimization Model

The first model finds the fraction of the HV which minimizes the expected cost of finding the familiar route. We obtain abstract expressions of the cost independently of search strategy, and find the start of search which minimizes the cost.

To an animal, the costs of searching are complex and include intrinsic and extrinsic factors such as time wasted, energy lost, predation risk, and exposure to the elements. Here we abstract the cost of searching to be any monotonically increasing function of the distance to a point to be found. Hence the further a point, the more costly the search for that point.

When a navigating agent searches for any point along a route, the overall cost must account for the cost of searching all possible points, weighted in some way. At the start of search, the agent does not know precisely where the familiar route is relative to itself. Hence whatever search strategy is used, it has no way to guarantee that it will find one particular part of the familiar route first. It could not, for example, decide to find the nearest point first, since it does not know where that point is relative to itself, nor can it maintain a noise-free course towards such a point even if it guessed correctly. It may even fail to recognize that point on the first encounter (for exponential detection law, see [31]). Thus, it is reasonable to suppose that there is some probability that any part of the familiar route may be found and recognized first over a large number of trials and random displacements. It is important to note that while the navigating agent may be searching for all points simultaneously with the goal of detecting any of them, on any particular trial, it is assumed that there is a single point which is detected first, after which it finds its way home.

For a set of points such as a familiar route, the expected cost function is modelled in two ways. Firstly, all points along the familiar route are assumed to contribute equally to the expected cost of search (Model 1a). In this way, the expected cost is found over all points along the familiar route. Secondly, the expected cost is found over the angular extent of the familiar route, subtended at the point of (ideal) start of search (Model 1b). In effect, the latter analysis provides a reweighting of points along the familiar route, reducing the contribution of points which are far away at the start of search.

The major advantage of these two ways of modelling the expected cost function is that they do not require a particular search strategy or distribution to be assumed. A disadvantage is that neither explicitly account for the probability distribution of points along the familiar route being found. The latter requires specification, at the very least, of a search distribution (see later).

### 2. Optimal Search Distribution Model

The second model finds the fraction of the HV corresponding to the maximum likelihood of finding any point along the familiar route. This is based on finding the prior distribution of the target, the familiar route. This is done by combining three results.

Firstly, from the well known optimal search theory of Koopman [32], the optimal search distribution for any target is the logarithm of its prior distribution. This assumes an exponential detection law. Secondly, cumulative PI random errors tend to Gaussian in the limit (e.g., [7], [8]) so that it is reasonable to approximate the uncertainty in searcher position as Gaussian. Thirdly, it is logically valid to consider searcher position uncertainty as equivalent to target position uncertainty, for the purpose of finding the optimal search distribution.

These three results are combined to give the optimal search distribution, given a familiar route, and therefore the position where maximum search effort should be placed. This position is compared with the optimal position(s) under the minimum expected cost models.

## Results

### 1. Cost Minimization Model

**Figure 3:**
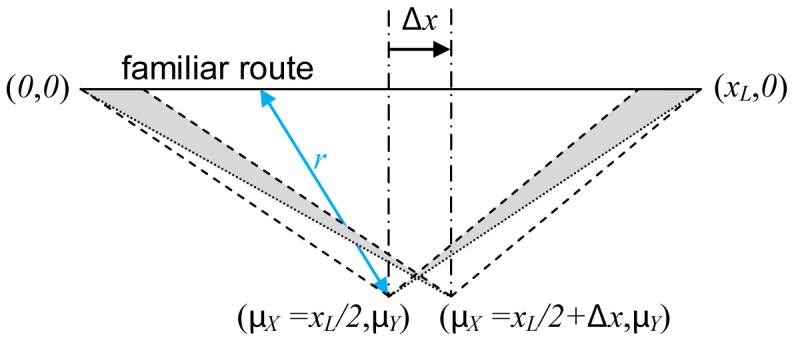
Geometric construction showing the distance (*r*) distribution between two possible locations to begin search, and the points along a familiar route (*L*) between the nest at 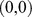 and the feeder at 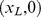. The two locations being compared are denoted 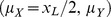 and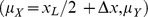. These represent two possible locations to begin searching for the familiar route, one exactly midway along a line parallel to the familiar route, and one slightly displaced from the midpoint. It is assumed the searcher has been displaced by some distance 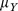 perpendicularly to the familiar route (NB: if 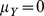, the searcher has found the familiar route). Congruent triangles (dashed lines) show equivalent distributions of distances to points along the familiar route, while the shaded areas show unequal distributions of distances (see text for details).

A geometric construction illustrating the following analysis is shown in Figure 3. The distance 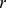 between the start of search, 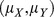, and the position of a familiar location 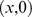 is
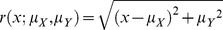
(1)


It is assumed that the cost of searching, 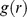, is some monotonically increasing function of 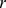. This assumption implies that the further a familiar location, the greater the cost to the ant of finding it. The quantitative contribution of each point along a familiar route to the overall cost of search depends on the relative proportion of time which is spent searching for that particular point, which depends both on the search strategy and distribution of target points.

From the midpoint i.e., 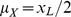, the expected (average) cost of searching for all points along the familiar route is
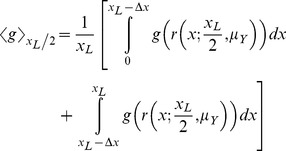
(2)


Shifted by some distance i.e., 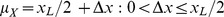, the expected cost of searching for all points along the familiar route is
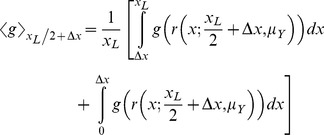
(3)


It can be seen that
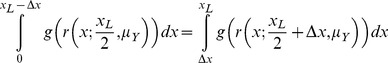
(4)because there is no change in the relative position between the search start and familiar route segment of interest (both shifted by 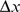). However,
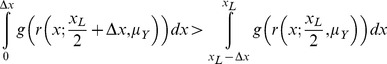
(5)since



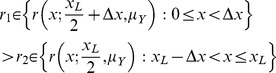
(6)This means 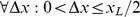,
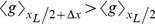
(7)


By symmetry of construction, equivalent arguments apply 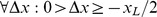. Therefore, the expected cost of search, 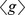, is minimized if and only if
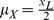
(8)


It is important to note that this result is independent of the search distribution or search algorithm.

A similar argument applies in considering the maximum cost of search rather than the expected cost of search. The monotonicity of 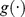 implies that
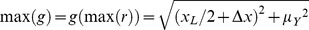
(9)so that
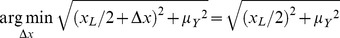
(10)if and only if 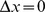 (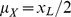). Hence the maximum cost of search is also minimized when search begins after homing for half the distance of the familiar route.

However, the minimum cost 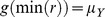 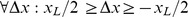, and is independent of 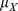 over the length of the familiar route. Hence the fraction of the HV used prior to searching does not affect the minimum cost of search. Nonetheless, there is no contradiction with the position minimizing the maximum or expected cost.

It can also be shown that the expected cost of search with respect to the angular extent of the familiar route is also minimized when 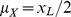 (Model 1b, Text S1, see also Figure S2). The consistency between Models 1a and 1b is not surprising because the underlying assumption in both models is that the cost of search increases with distance. Since the distance to the furthest end of the familiar route increases with 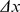, points of the familiar route contributing to the most costly search are minimized by setting 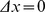. These results support the hypothesis that the search initiation point which minimizes search cost is relatively insensitive to the search distribution, and should be midway along the familiar route according to PI.

The validity of the above theoretical assumptions is illustrated using three simple search algorithms, in simulation (Figures S3, S4, S5, S6, Table S1, S2). Firstly, the random displacement immediately prior to homing (red dashed lines in Figures S3A, S4A and S5A) combined with sensorimotor noise in the execution of any search strategy result in a distribution of first detections over the entire familiar route (Figures S3B, S4B and S5B). This is true even if the familiar route is detected on every encounter (perfect detection). Secondly, expressed as the average number of steps needed to detect the familiar route, the average cost of search increased monotonically with the distance to a point along the familiar route (Figures S6A, S6C and S6E). Most importantly, the average cost of search was minimized when 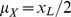, irrespective of whether the ‘cost’ was the mean number of steps until first detection of the familiar route, or the ‘cost’ was the probability of failure of detecting the familiar route within a predefined number of search steps (Figures S6B, S6D and S6F, including insets).

### 2. Optimal Search Distribution Model

The previous analysis did not define a particular search distribution to most efficiently find the familiar route. The problem modelled next is an extension of [32] and similar assumptions are made (see also [18]). Some key results from Koopman [32] relevant to the current work are outlined below.

Assuming an exponential detection law, the optimal search distribution for a point target was proven to be the natural logarithm of the prior distribution of the target [31]. For a Gaussian prior distribution, the optimal search density function is therefore an inverted parabola. The exponential detection law was derived assuming that target recognition is imperfect, so that for some small search effort, 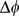
_,_ allocated to the vicinity of the target, there is some probability, 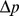, of detecting the target, and that 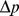 is constant for each equivalent search effort applied to the vicinity of the target, independent of previous allocations of search effort at that location. In the limit, 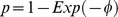. See Koopman [31], [33] for more detailed analytic treatment of the target detection process, and underlying assumptions and limitations of this model.

The original optimal search problem was defined for a point target, and where the searcher’s position is known at all times. This concept can be extended to a target which is an entire route in 2D space, and where the searcher’s position is uncertain. The route itself may be considered as a set of points, each of which is detectable by the searcher according to the same detection function.

Consider firstly the simplified problem of searching for either of two point targets in 1D, denoted 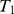 and 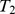, separated by a fixed distance *r*. The search succeeds if either target is found. This is equivalent to two small targets which are fixed in allocentric space, but due to the searcher’s uncertainty about its own position, the position of either target relative to the searcher is uncertain, but the uncertainty distributions are perfectly correlated (since 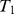 and 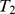 are fixed in their relative positions).

**Figure 4:**
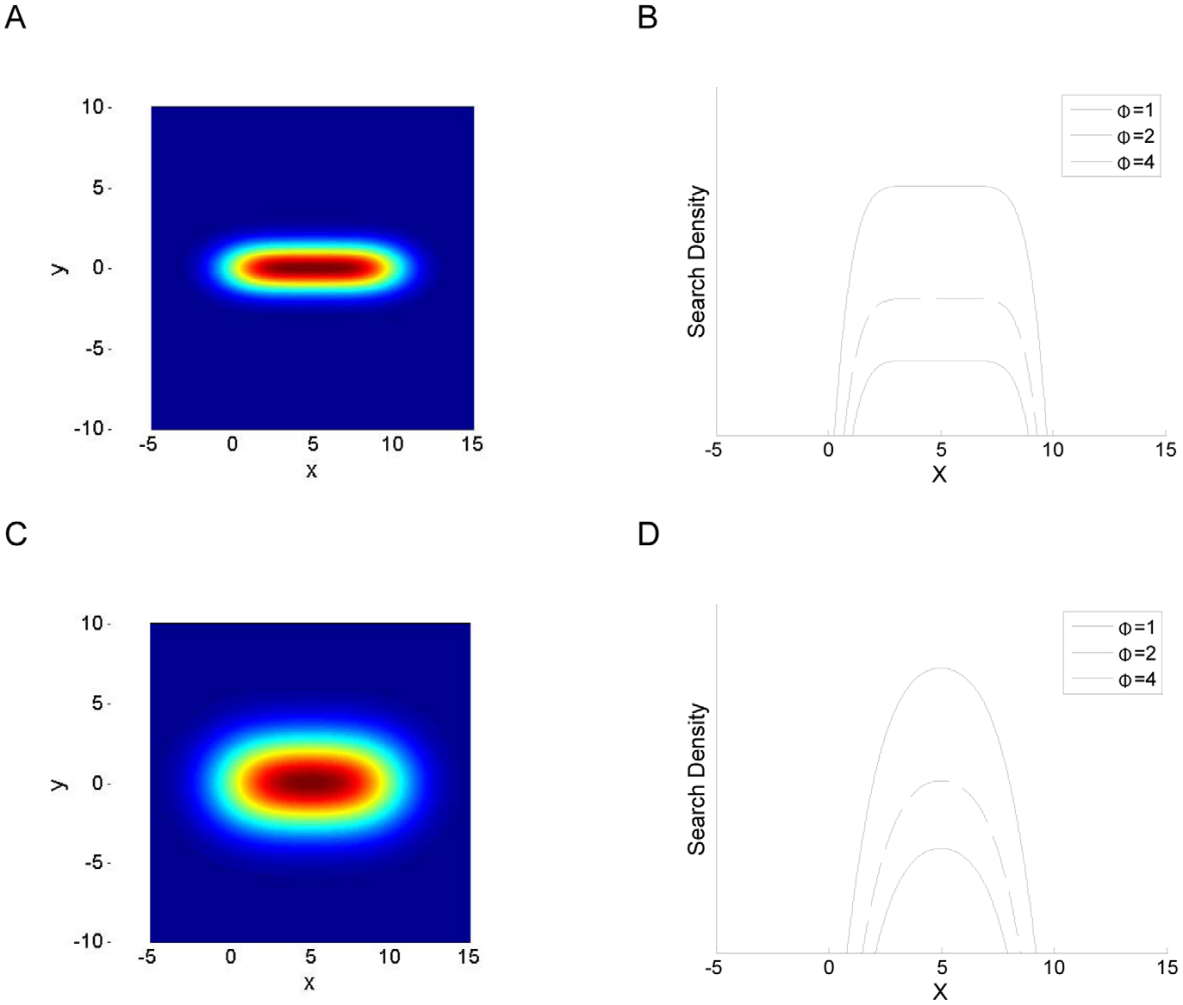
Optimal search distributions for a familiar route. A. Probability density heat map for a familiar route (straight line segment) between 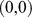 and 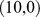, with Gaussian uncertainty whose standard deviations 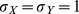. B. The marginal optimal search density for a total search effort, 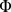, of 1, 2 or 4 arbitrary units. Note that the integral under each search density function equals the total search effort [32]. C and D as per A and B, respectively, with 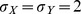 (twice the width of the uncertainty distribution as A).

Suppose the prior distribution of the targets, i.e., 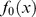 and 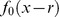 are known to the searcher. The optimal search problem becomes one of finding the search density function which maximizes the probability of detecting either 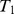 or 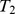 for any planned search horizon (predetermined total search effort 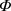 - illustrated in Figure 4, see also [32]).

From the searcher’s perspective, the above problem is similar to the original problem of finding a single target whose position is uncertain. Firstly, for the purpose of target search, the relative uncertainty between searcher and target can be considered to be independent of whether positional uncertainty is associated with the searcher or the target. That means the uncertainty in allocentric position of the searcher (due to cumulative PI errors) can be considered to be equivalent to the uncertainty in position of the target *relative* to the searcher. Secondly, it is assumed here that search success does not depend on *which* target is found, only that *a* target is found (which is true if the aim of search is to find any point along a familiar route).

The probability of 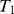 being at 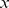 is 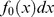, while the probability of 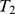 being at 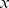 is 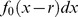. Note that 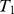 and 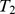 can never be at 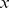 simultaneously as they are always separated by 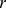. The two-target problem differs from the one-target problem in the following way. Suppose a search distribution is fully executed even if a target is found early. For one target, the probability of detecting that target is equivalent to the expected number of targets detected per search, always between zero and one. For two targets, the expected number of targets detected per search may exceed one, under the assumption that pre-allocated search is executed fully. In other words, in some searches, both targets are detected within the pre-allocated search effort. In practice, the searcher may abandon search once the familiar route is found.

Strictly, maximizing the expected number of targets detected in the two-target problem is not mathematically equivalent to optimizing the probability of detecting either target. For example, if the probability of detecting both targets is increased without affecting the probability of detecting individual targets, the expected number of targets detected is increased, without increasing the probability of detecting either target. However, by maximizing the expected number of targets detected, it is likely that a realization of the search distribution will detect a target early, providing benefit to the searcher. Furthermore, this formulation of the problem provides a simple mathematical solution for the optimal search effort distribution which can be found directly following Koopman [32].

From the earlier example, the combined density function is,
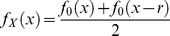
(11)which is the probability density of either 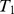 or 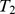 being at 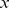. Note the normalization factor is required to preserve the property that 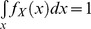. Normalization to preserve the properties of a probability density function is convenient when this result is generalized as a convolution (see later). However, an alternative formulation without normalization can also be used, i.e., 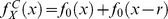. Intuitively, 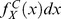 may be considered as the frequency of targets at (or close to) 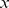. Hence the total frequency of two targets over all space 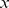 is two, i.e., 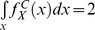.

An analogous 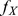 can be found for any number of points 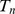. Next consider that a line segment (representing a familiar route) is a continuum of points. For a familiar 1D route along segment 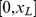, the density function is simply the convolution of a uniform distribution of points along the line segment, with the uncertainty distribution. Simplified, it is
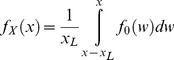
(12)


More generally, the convolution may be written as
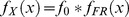
(13)where 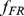 is the distribution of points along the familiar route.

For instance, if 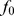 is Gaussian, and 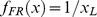 along the familiar route as above,
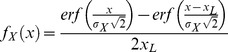
(14)


Similarly if 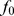 is Gaussian in 2D, and assuming a familiar route along the straight line segment between 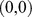 and 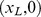,
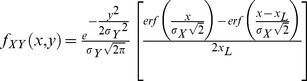
(15)


Figure 4 shows two examples of the optimal search distribution for different uncertainty standard deviations.

The bivariate Gaussian is a good approximation of the positional uncertainty distribution of a PI system which has a compass [7], [8]. However, the precise distribution of the positional uncertainty is not critical for the model results to hold. From Eq 12, as long as uncertainty and search properties are such that the optimal search distribution 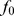 is symmetric and unimodal for any point target, 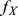 is also unimodal with global maximum at 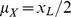.

Following Koopman [32], the optimal search distribution is:
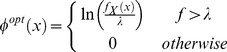
(16)where 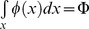 is the total search effort. Graphically, the value of the Lagrange multiplier 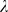 may be found by sliding a horizontal line up or down until the area between the logarithm of 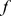 and the logarithm of 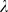 is exactly 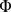. For a Gaussian 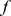, the optimal distribution of search effort, 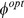 is therefore an inverted parabola. Note that 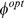 is independent of constant scaling of 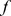, so that using 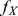 or 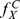 yields the same result for 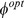. For further details see Koopman [32], [33].

Two important consequences of Eq 16 are as follows. Firstly, if 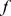 has a global maximum at 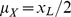 then so does the optimal search distribution 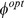. Secondly, total search effort 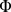 may be increased at any time, without affecting the optimality of the search up to that point in time. This is because the effect of increasing 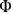 is equivalent to lowering 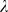, which means all previous search effort is still included in the new optimal search distribution. However, if the total search effort is not pre-allocated, there is only one location which is guaranteed to be part of any optimal search distribution, which is the global maximum of 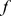, i.e. 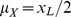. Therefore, this is an optimal position to begin search, irrespective of the total search effort which will eventually be exerted.

It is worth noting that the arguments presented above may be generalized to alternative target detection models. For instance, Wehner & Srinivasan [15] assumed that the ideal search density function should match the prior uncertainty distribution rather than its logarithm. Under this assumption, the search effort distribution should also be maximal when 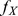 is maximal, which occurs when 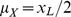, under the assumptions stated earlier.

## Discussion

The distance individual ants travel relying on their path integrator in an unfamiliar terrain decreases from landmark-poor saltpans, to landmark-rich desert scrub and to landmark-dense rainforest. The fractional read-out of HV information shown by ants occupying landmark rich and dense habitats may be related to the range over which these ants know the visual scene around their nests. Here, we used two theoretical analyses to find the optimal start of search, given an accurate HV obtained through PI, and a familiar route. The first analysis assumed that the cost of search is monotonically dependent on the distance to the search target. For a homogeneous set of points along the familiar route, the optimal position was shown to be the midpoint of the familiar route according to the PI system. The second analysis assumed that the start of optimal search should begin at the mode of the optimal search density function. For any symmetrical unimodal positional uncertainty distribution, the modal position is the midpoint of the familiar route according to the PI system. The start of search according to both the cost minimization and optimal search distribution models is in agreement, and corresponds to the midpoint of the familiar region according to the PI system.

As a first approximation, the familiar region was assumed to be a thin, linear region extending from the nest. This scenario is a geometric approximation of the experimental conditions where *Melophorus bagoti* trained to a feeder 20 m away from the nest returned home in a narrow 0.5 m wide corridor of landmarks (see Fig. 3 in [19], Fig. 2b in [21] for landmark corridors encountered during natural foraging). Much like observations in *M. bagoti* [29], the optimal ant should run off about half of its HV prior to initiating search (Figure S1B).

Conversely, the theoretical results predict that an ant which runs off most of its HV prior to searching is likely to have a relatively small familiar range compared with the foraging range (Figure S1A). This is consistent with observations in *Cataglyphis fortis* where its nest is typically a tiny hole in a large, cue-poor and wide open environment.

Recent evidence from *M. bagoti* that inhabits landmark-poor habitats indicates that upon displacement they travel nearly 70% of their HV [30]. Does this mean that *M. bagoti* ants dynamically optimise their homing strategy depending on the available landmark information? When both the foodward and nestward routes of *M. bagoti* ants were restricted to linear tunnels ants were guided by HV information over nearly the full home distance [29]. This is most likely because the visual context during both the outward and nestward trips was similar. If the homing mechanism of *M. bagoti* is truly dynamically optimized, then it should be testable in an experiment where ants familiar with a long landmark corridor (e.g., 20 m) is provided later with a food source at midway (i.e., 10 m) for a few trials. If these ants are displaced, a dynamically optimized homing system should begin search immediately to minimize expected cost and maximize expected probability of finding the familiar route of 20 m. Alternatively, if *M. bagoti* runs off approximately half its HV (i.e., about 5 m) prior to searching, this may suggest a strategic optimization for commonly encountered conditions rather than dynamic adaptation from one foraging trip to the next (Figure S1C; see [30]).

In contrast, it is possible that in some landmark-rich environments the familiar range extends beyond the typical foraging route. This might explain the observation that tropical rainforest ants such as *Gigantiops destructor* travel only about 5–25% of the true distance towards home before starting their search [27]. If the familiar range is close to double the distance between nest and foraging zone, then the foraging zone is close to the midpoint along the familiar region. Hence the optimal search should begin almost immediately after release, as observed. It is also possible that *G. destructor* begins homing along the theoretical feeder-to-nest vector for an obligate distance, e.g. 0.5 m [27], rather than as a fraction of the HV. Such a result would argue against the use of a fractional HV as a general adaptive mechanism across all ant species. To test this possibility, it is necessary to collect more information on the start of search from a wide range of nest-feeder distances in the natural environment of *G. destructor*. It will be equally important to test whether these ants rely on their HV when their foodward and nestward trips are restricted to linear channels.

Search is a crucial component in the ant’s navigation toolkit (e.g., [15], [34]). One possible trigger for the activation of search may simply be that some cumulative level of unfamiliarity is reached, independent of the HV or size of the familiar region. Computationally, this could be mediated by a familiarity network (e.g., [35]) which has learnt views along the familiar route. When displaced, an accumulation of novel views could perhaps cross some threshold for initiating search. Under this hypothesis, ants should travel different distances along the HV before beginning their search, depending on visual unfamiliarity. Experimentally, however, the HV distance in both ‘slightly familiar’ (ants displaced laterally from the nest-feeder route) and ‘unfamiliar locations’ (ants displaced to distant locations) have been shown to be similar [21], arguing against a simple unfamiliarity threshold model. More work is needed to rigorously quantify the view differences between the ‘slightly familiar’ and ‘unfamiliar locations’.

Although this work focused on the initiation of search rather than the search algorithm itself, it is clear that the search strategy affects the cost and effectiveness of search. Vickerstaff & Merkle [36] recently showed that a Bayesian model of systematic search for home is better able to cope with continually accruing positional uncertainty than other models. It may be possible to extend the Vickerstaff and Merkle model to incorporate familiar routes, so that predicted search path characteristics may be tested experimentally.

The home of a central place foraging animal is a special location, often explored more frequently than other foraging areas. The ease of detection and/or value of detection may vary according to the position along a familiar route. If known, these functions of position may be incorporated explicitly into the formulation of the optimal effort distribution, using a change of variable method [32].

Qualitatively, it would be expected that if the value and ease of finding home is significantly higher than other parts of the familiar route, search initiation should be biased towards home. On the other hand, since positional uncertainty increases for the entire duration away from the familiar route, delaying the initiation of search may increase the total number of steps needed to find the familiar route, partially negating the benefit of the former. Finally, the familiarity of the route, and hence ease of detection of the route, may vary depending on the orientation of the animal, not just its position [37]. Combining detailed experimental and theoretical studies of these factors will be required to determine how an ant may fine tune its search initiation point.

To further complicate matters, experimental evidence suggests that the available visual information may directly influence the search strategy *per se*. Individual homing ants (*M. bagoti*) caught close to the nest (zero-vector ants), when displaced far away from their familiar region, search more or less symmetrically around the release location [21], [38]. In contrast, zero-vector ants released only 10 m laterally to their familiar route engage in a search which shows a clear bias towards the nest (Fig 6 in [21]), suggesting that familiar visual cues influence the search trajectory. Similarly, when animals with full vector information are displaced 10 m laterally from their familiar route, they run off nearly half their HV and then engage in a progressive search with a bias towards the nest (Fig 5 in [21]). To fully understand the complex interplay between PI and search, it is therefore critical to characterise the complete range of search strategies along with the information content of the environment, together with the state of the PI system.

The experimental and theoretical results described here also have implications on the nature of the neural networks subserving path integration. From a computational perspective, there needs to be an accurate path integration system and, if the familiar route is to be (approximately) bisected, then there needs to be metric properties associated with the familiar region. It is unclear at present whether a decentralized neural architecture such as Cruse & Wehner [34] suffices, or whether a single coherent representation of the spatial world is required. Possible neural models of path integration able to replicate fractional HV use are currently under investigation.

## Supporting Information

Figure S1 **The familiar region affects the optimal homing strategy.** A. When the foraging journey (blue) extends beyond the familiar region (grey), the optimal homing animal follows the direction of its HV (dotted line) to the middle of the familiar range, from where it finds its home directly. A sudden displacement (red dashed) results in the homing animal unable to reach the familiar region following running off nearly 100% of its HV (red solid line), at which point it initiates search. B. As per (A) but with a typical foraging journey within the familiar range. The optimal homing animal follows the direction of its HV to the middle of the familiar range, which now corresponds to just under halfway along the full HV. C. Proposed experiment to test whether the fraction of HV used adapts dynamically to the familiar region or is tightly coupled to the magnitude of the HV at the start of homing.
(TIFF)

Figure S2
**Geometric construction showing angular extent of different segments of the familiar route 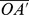, relative to 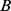 and 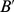.** These represent two possible locations to begin searching for the familiar route, one exactly midway along a line 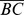 parallel to the familiar route, and one displaced by 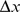 from the midpoint respectively. Note that 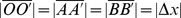. Using the convention of Fig. 3, 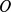 is at 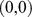, 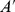 is at 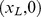, 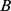 is at 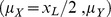, and 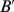 is at 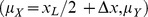.(TIFF)

Figure S3
**Correlated random walk search model.** A. Three random search trajectories (rows) are shown for four different fractional HVs (columns) used. The home (light red dot), familiar route (black line), last known location (cyan dot), random displacement (red dashed line), fractional use of the ideal HV (solid red line) are superimposed. B. Top left panel shows the frequency histogram of positions of first detection of the familiar route, pooled from the frequency distributions at each of eleven ideal search initiation position 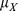
(other panels). All bin widths were 0.2 linear units.(TIFF)

Figure S4 **Archimedean spiral search model – otherwise as per Figure S3.**
(TIFF)

Figure S5 **Modified** ***Cataglyphis*** **search model – otherwise as per Figure S3.**
(TIFF)

Figure S6
**Cost of search.** Mean ± s.e.m. of the number of steps needed to detect the familiar route as a function of the distance *r* between the start of search and detection point, pooled over all trials in bins of 0.2 linear units, are shown for the correlated random walk search (A), Archimedean spiral search (C) and modified *Cataglyphis* search (E). Mean ± s.e.m. of the number of steps needed to detect the familiar route as a function of the ideal search initiation position 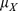, are shown for the same search models respectively in B, D, and F. Insets show the probability of failing to detect the familiar route following 10^5^ search steps. Using these models it is not possible to assign a probability of failure to a particular radial distance since no particular point along the familiar route can be associated with the failure to detect the familiar route.(TIFF)

Table S1 Search model descriptions.
(TIFF)

Table S2 Simulation parameters common to all search models.
(TIFF)

Text S1 Model (1b): Cost Minimization.
(DOC)
