# Supplementary figures and images for: Ant Navigation: Fractional Use of the Home Vector
Source: PLoS One. 2012 Nov 29;7(11):e50451. doi: 10.1371/journal.pone.0050451 (PMC3510198; doi:10.1371/journal.pone.0050451)

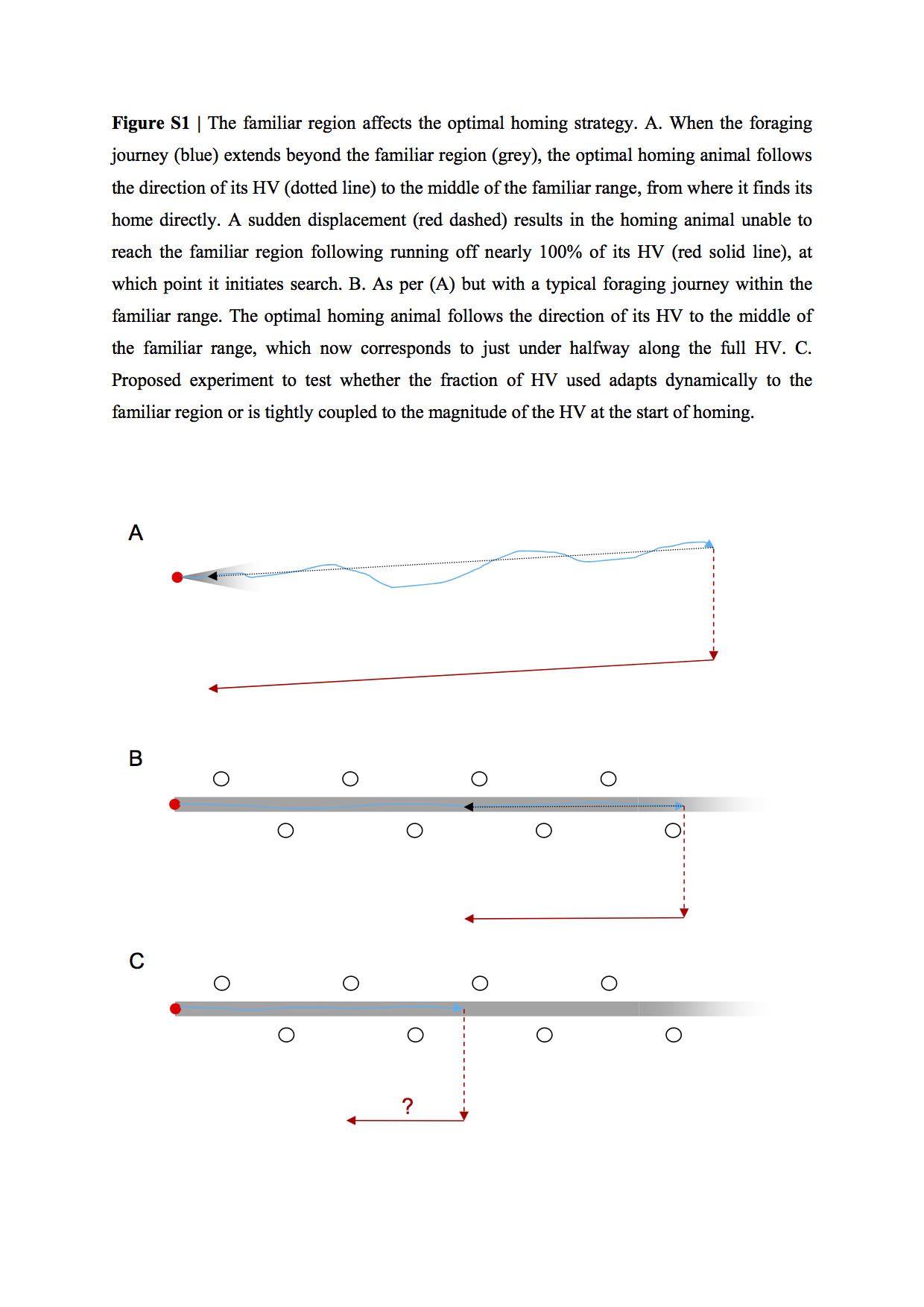

Supplement: Figure S1 — The familiar region affects the optimal homing strategy. A. When the foraging journey (blue) extends beyond the familiar region (grey), the optimal homing animal follows the direction of its HV (dotted line) to the middle of the familiar range, from where it finds its home directly. A sudden displacement (red dashed) results in the homing animal unable to reach the familiar region following running off nearly 100% of its HV (red solid line), at which point it initiates search. B. As per (A) but with a typical foraging journey within the familiar range. The optimal homing animal follows the direction of its HV to the middle of the familiar range, which now corresponds to just under halfway along the full HV. C. Proposed experiment to test whether the fraction of HV used adapts dynamically to the familiar region or is tightly coupled to the magnitude of the HV at the start of homing. (TIFF) [file pone.0050451.s001.tiff]

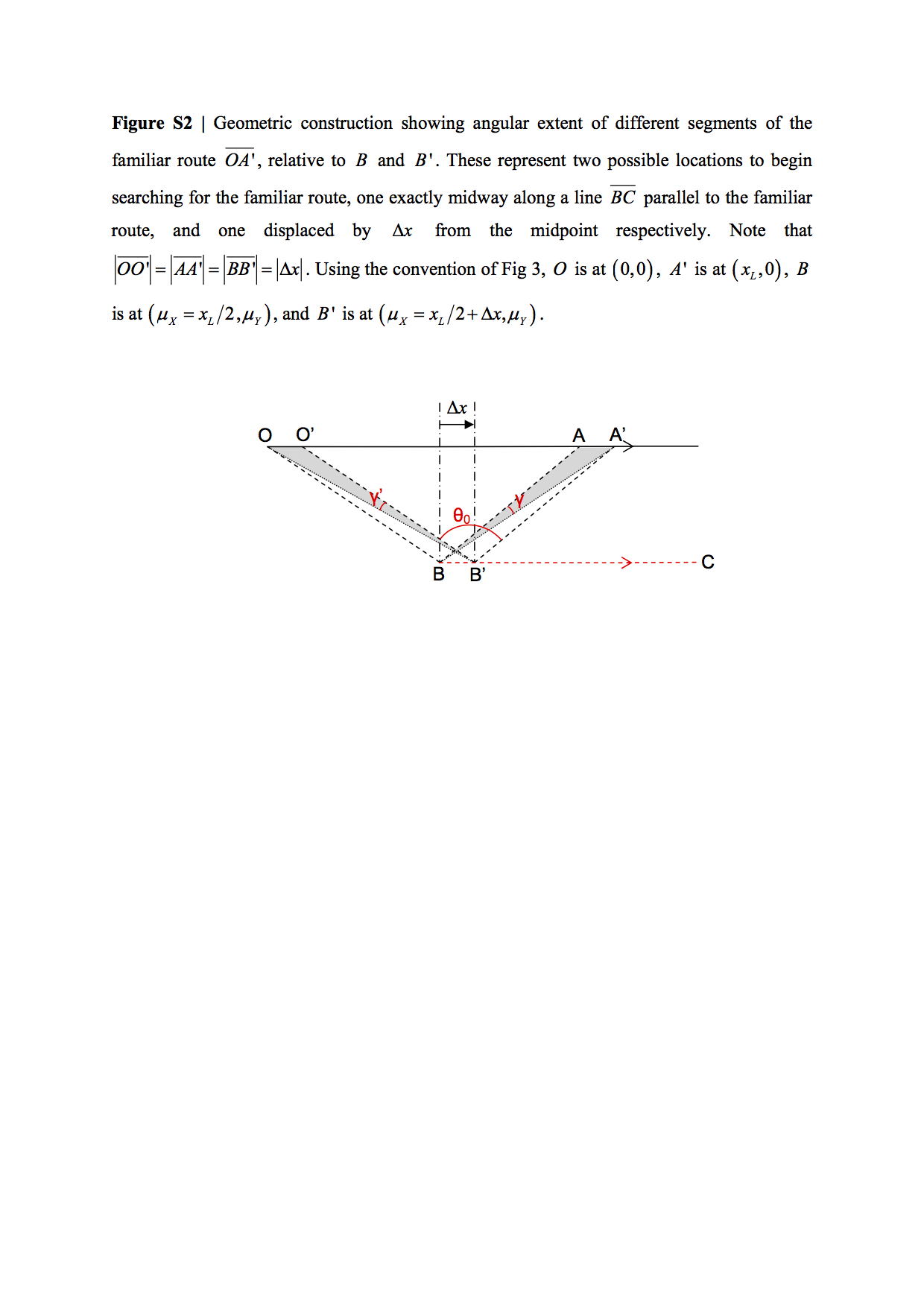

Supplement: Figure S2 — Geometric construction showing angular extent of different segments of the familiar route , relative to and . These represent two possible locations to begin searching for the familiar route, one exactly midway along a line parallel to the familiar route, and one displaced by from the midpoint respectively. Note that . Using the convention of Fig. 3, is at , is at , is at , and is at . (TIFF) [file pone.0050451.s002.tiff]

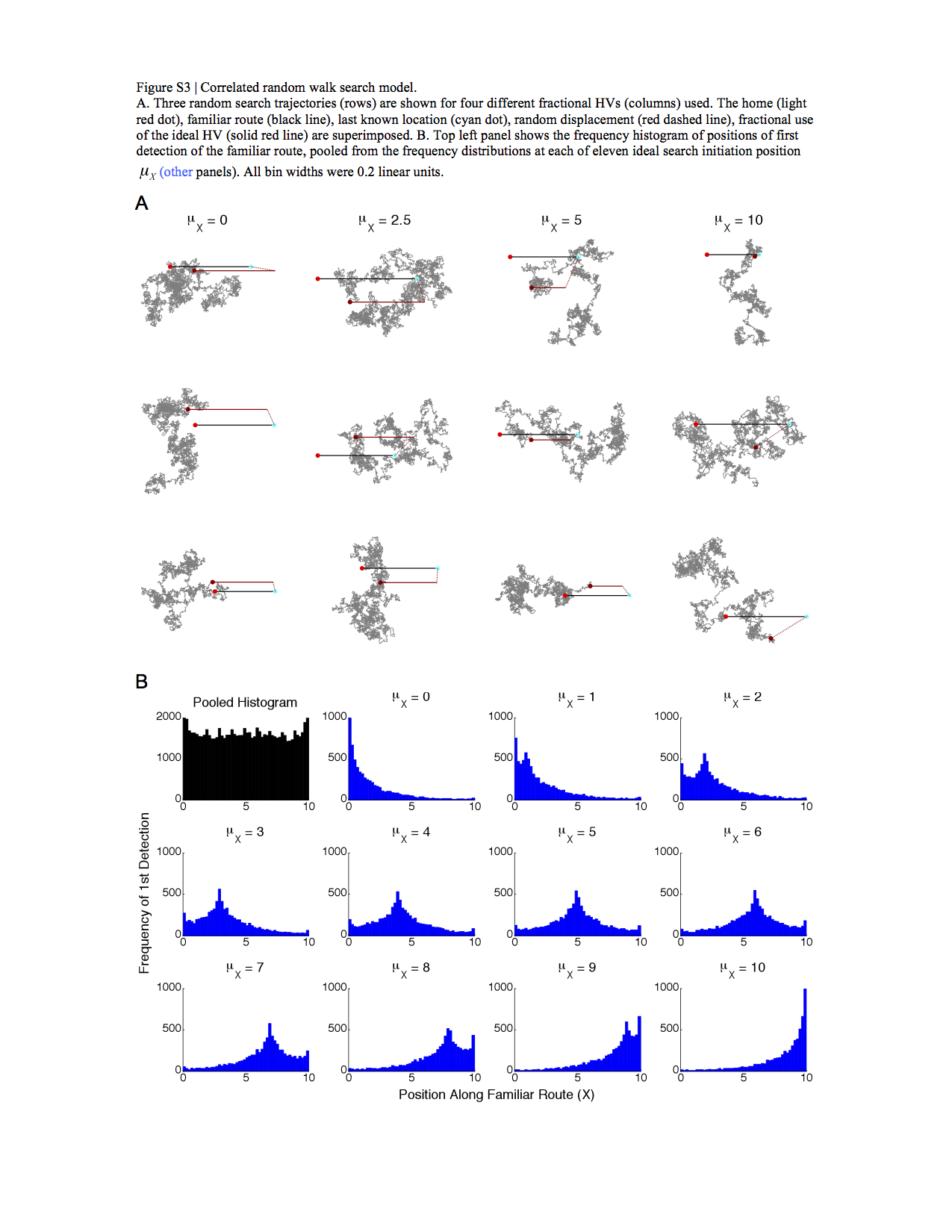

Supplement: Figure S3 — Correlated random walk search model. A. Three random search trajectories (rows) are shown for four different fractional HVs (columns) used. The home (light red dot), familiar route (black line), last known location (cyan dot), random displacement (red dashed line), fractional use of the ideal HV (solid red line) are superimposed. B. Top left panel shows the frequency histogram of positions of first detection of the familiar route, pooled from the frequency distributions at each of eleven ideal search initiation position (other panels). All bin widths were 0.2 linear units. (TIFF) [file pone.0050451.s003.tiff]

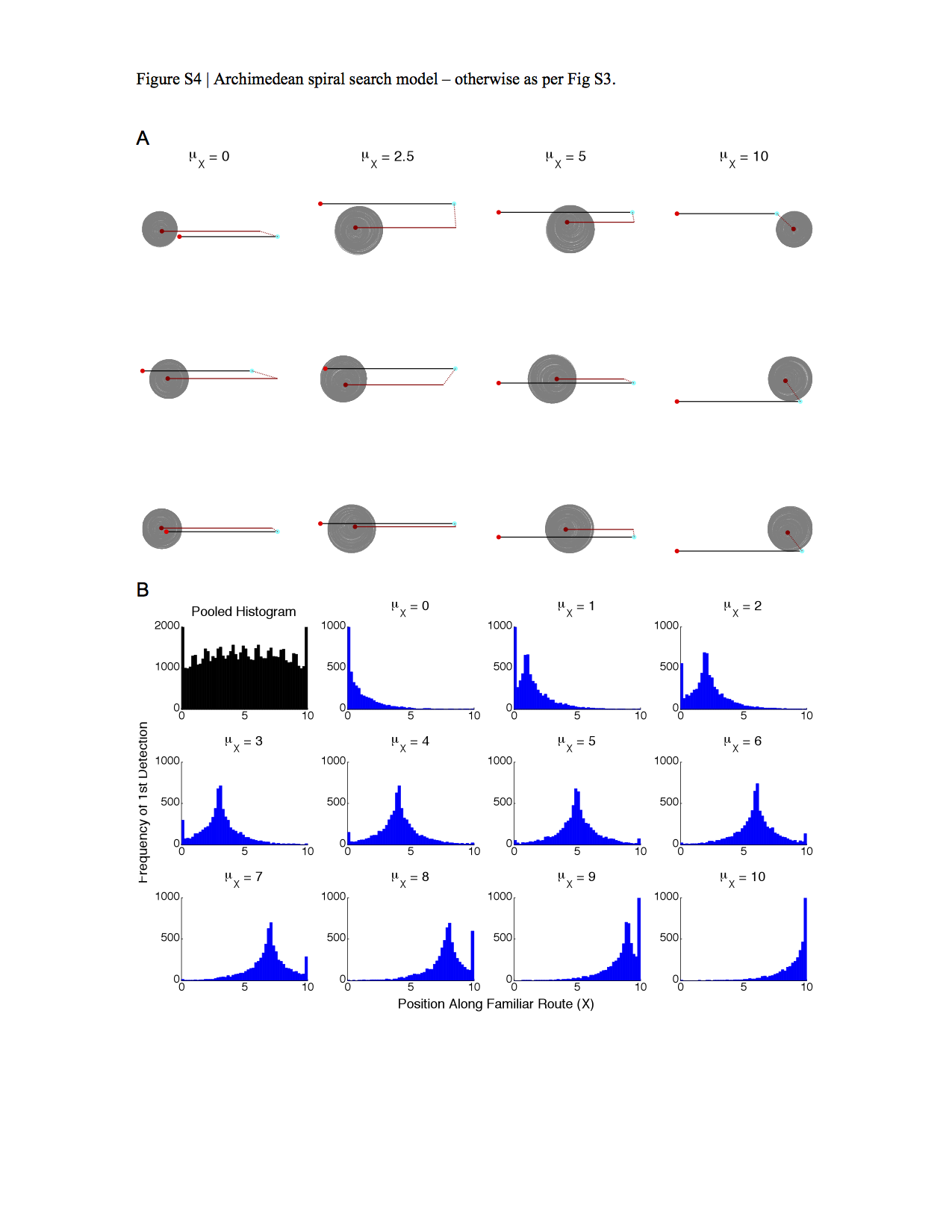

Supplement: Figure S4 — Archimedean spiral search model – otherwise as per Figure S3. (TIFF) [file pone.0050451.s004.tiff]

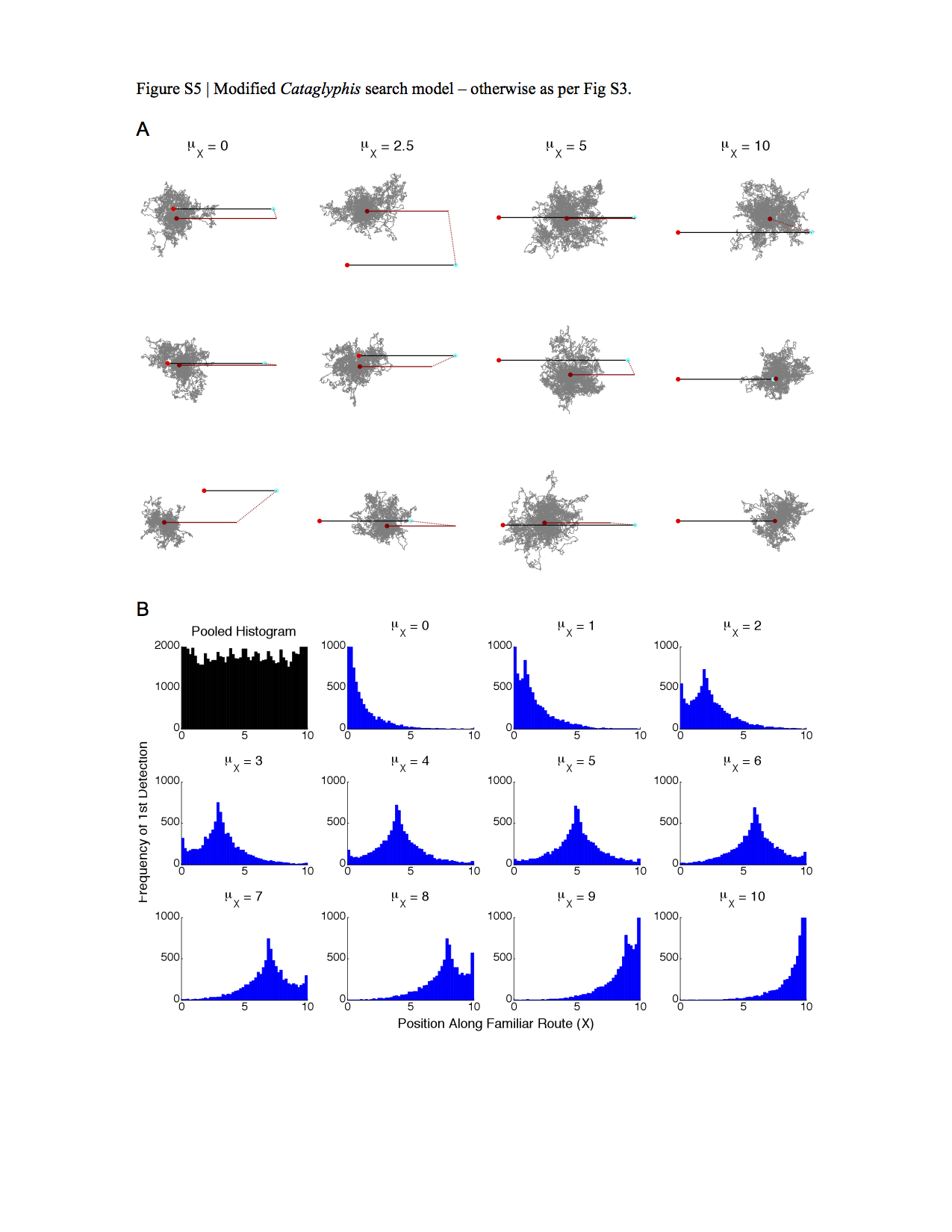

Supplement: Figure S5 — Modified Cataglyphis search model – otherwise as per Figure S3. (TIFF) [file pone.0050451.s005.tiff]

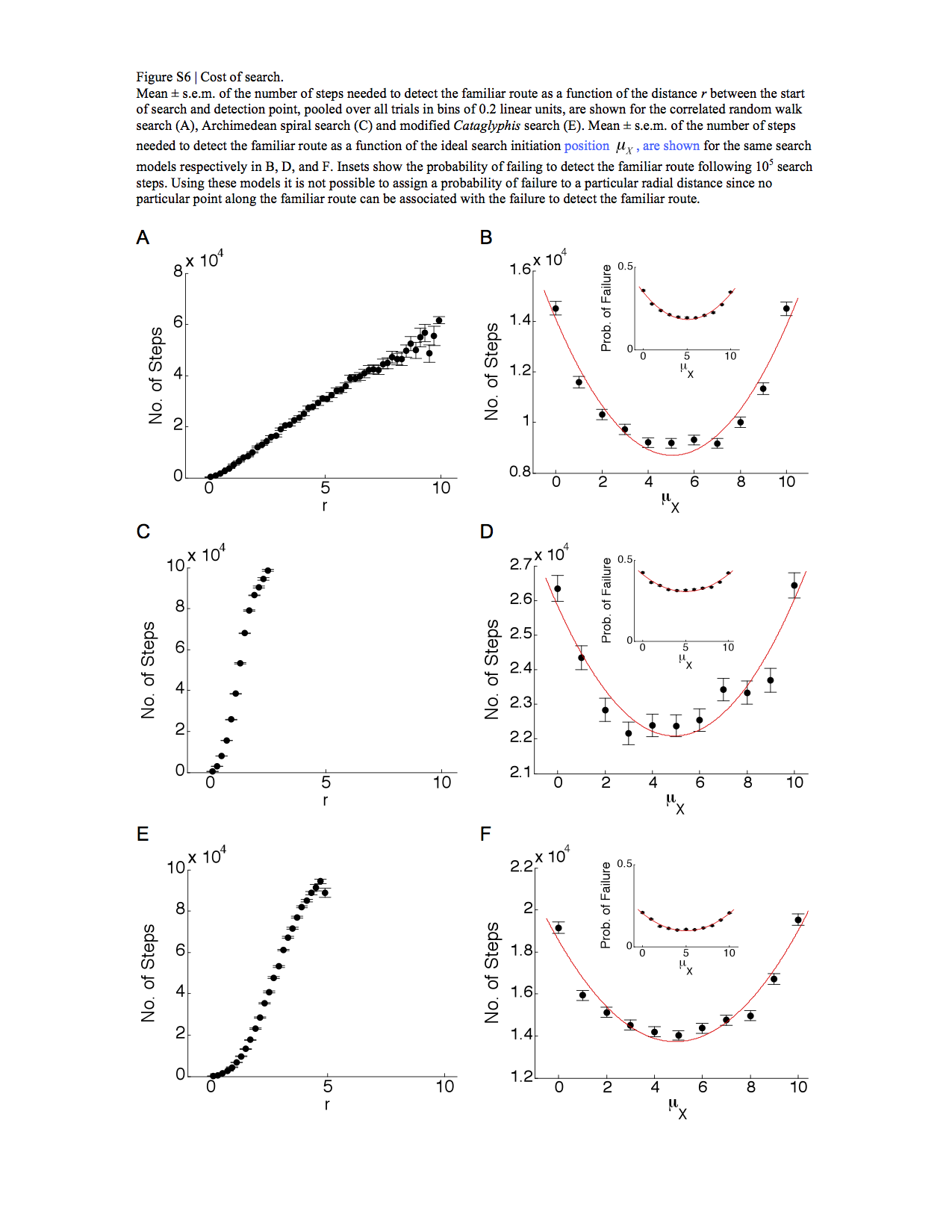

Supplement: Figure S6 — Cost of search. Mean ± s.e.m. of the number of steps needed to detect the familiar route as a function of the distance r between the start of search and detection point, pooled over all trials in bins of 0.2 linear units, are shown for the correlated random walk search (A), Archimedean spiral search (C) and modified Cataglyphis search (E). Mean ± s.e.m. of the number of steps needed to detect the familiar route as a function of the ideal search initiation position , are shown for the same search models respectively in B, D, and F. Insets show the probability of failing to detect the familiar route following 105 search steps. Using these models it is not possible to assign a probability of failure to a particular radial distance since no particular point along the familiar route can be associated with the failure to detect the familiar route. (TIFF) [file pone.0050451.s006.tiff]

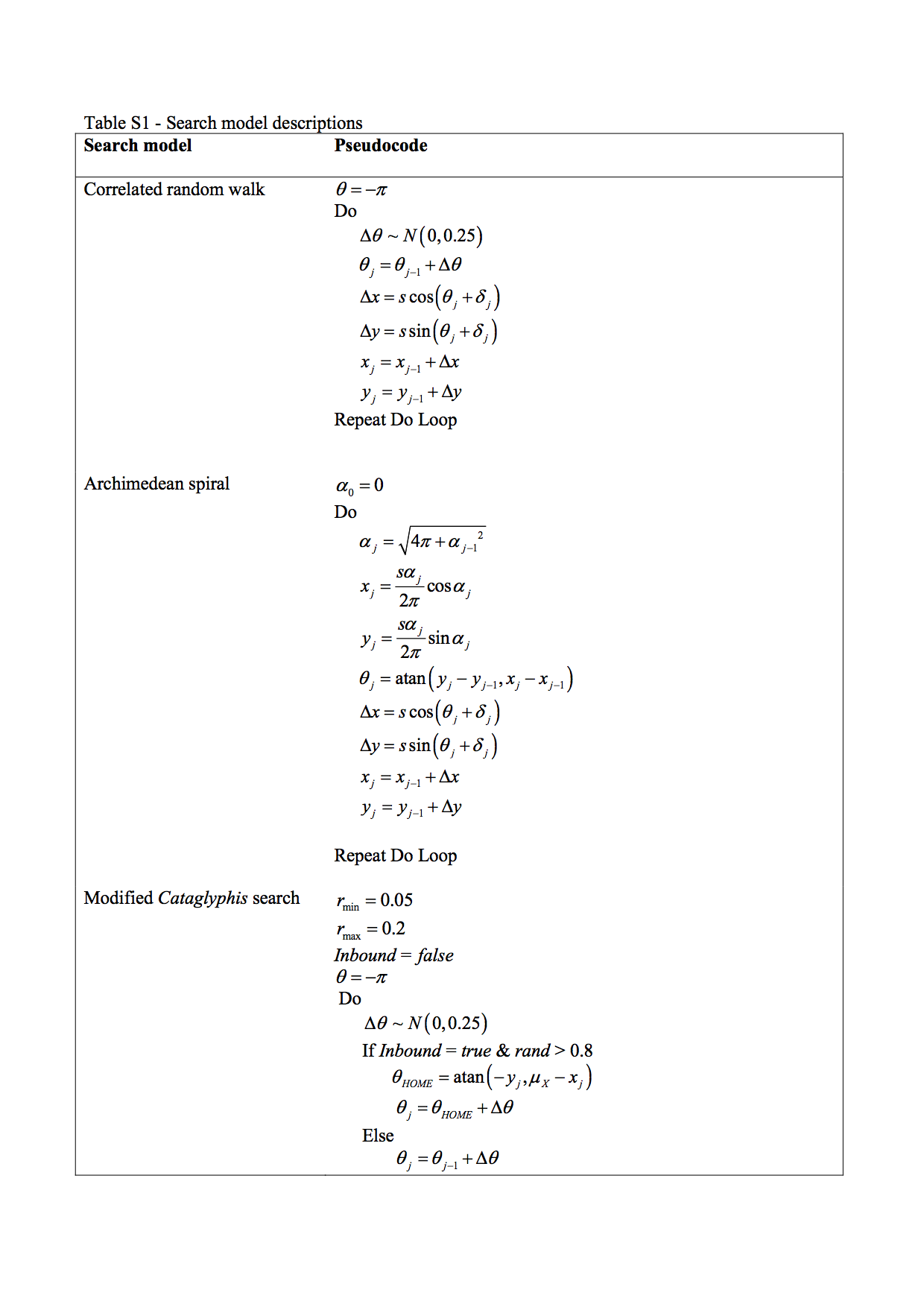

Supplement: Table S1 — Search model descriptions. (TIFF) [file pone.0050451.s007.tiff]

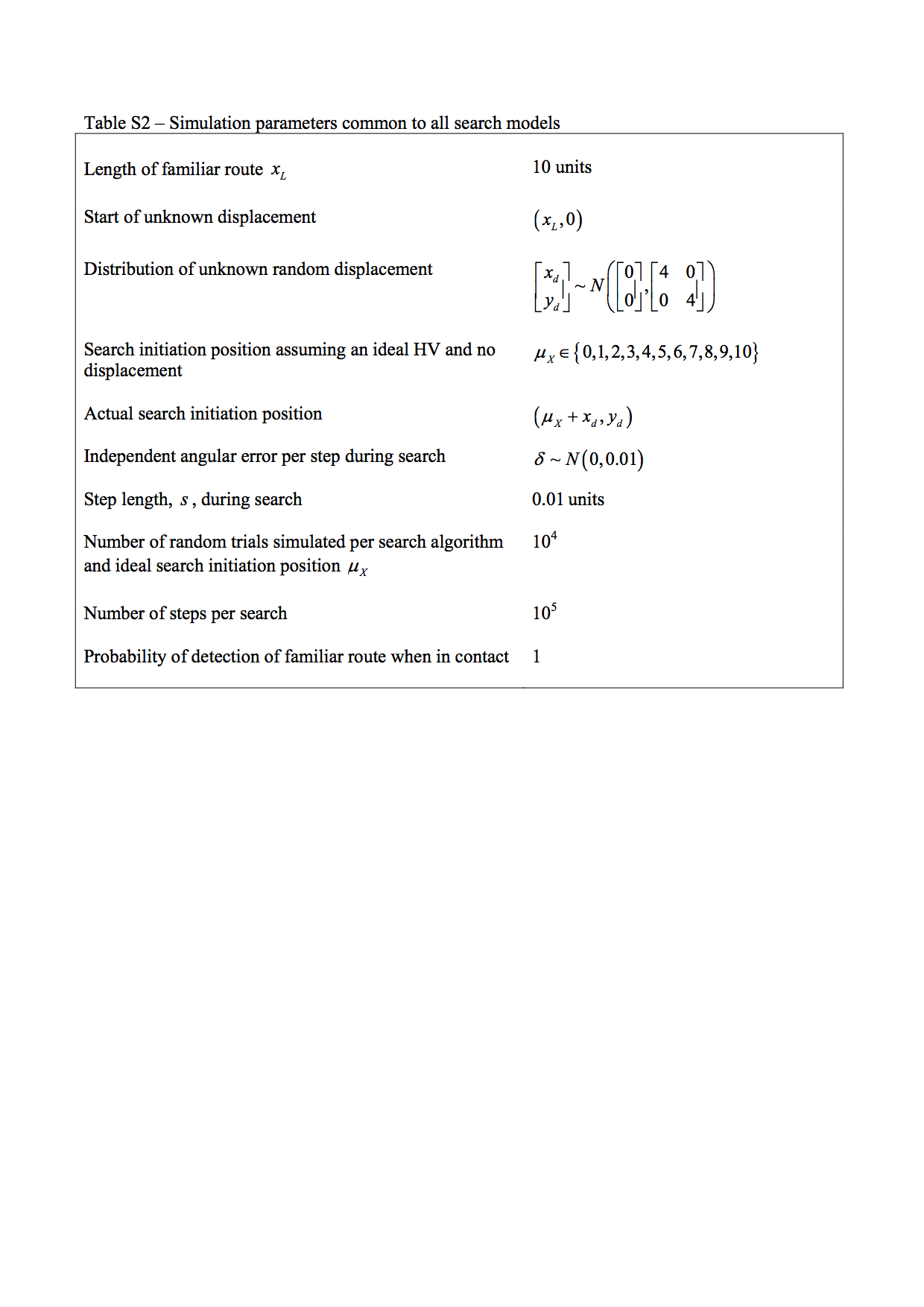

Supplement: Table S2 — Simulation parameters common to all search models. (TIFF) [file pone.0050451.s008.tiff]
