# Supplementary material for: Ant Navigation: Fractional Use of the Home Vector
Source: PLoS One. 2012 Nov 29;7(11):e50451. doi: 10.1371/journal.pone.0050451 (PMC3510198; doi:10.1371/journal.pone.0050451)
Supplement: Text S1 — Model (1b): Cost Minimization. (DOC) [file pone.0050451.s009.doc]

**Text S1**

**Model (1b): Cost Minimization**

In this section, the cost of searching is weighted uniformly over the angular extent of the familiar route at the start of search. This is in contrast to the earlier analysis which weighted the cost uniformly over the linear spatial extent of the familiar route.

For convenience, the distance $r$ between the start of search, $\left(\mu_{X},\mu_{Y}\right)$, and the position of a familiar location $\left(x,0\right)$ is defined $\forall\theta:\arctan\left(-\mu_{Y},x_{L}-\mu_{X}\right)\leq\theta\leq\arctan\left(-\mu_{Y},-\mu_{X}\right)$ as

$$r\left(\theta;\mu_{X},\mu_{Y}\right)=\frac{-\mu_{Y}}{\sin\theta}$$

where arctan(y,x) is the 4-quadrant arctangent function. Note that these conventions are suitable for $\mu_{Y}<0$ (Figure S2). It is clear from the construction of Fig S2 that the angular extent of the familiar route from $\left(\mu_{X},\mu_{Y}\right)$ is maximum when $\mu_{X}=x_{L}/2$, because

$$\angle OB'A'<\angle OBA'=\tan^{-1}\left|\frac{2\mu_{Y}}{x_{L}}\right|$$

This property itself may be a potential advantage for beginning a search at $B$ as compared to $B'$ since the familiar route occupies a wider azimuthal space at $B$. However, without specifying a search strategy *a priori*, expected cost minimization is not guaranteed by maximizing total angular extent.

The expected cost of search with respect to angular extent of the familiar route is found as follows. Since $\Delta OBA\equiv\Delta O'B'A'$ (congruent triangles), $\angle OBA=\angle O'B'A'$, whose angular magnitude is denoted $\theta_{0}$ in Fig S2. Since the distribution of distances between $\overline{OA}$ and $B$ is exactly the same as between $\overline{O'A'}$ and $B'$, the expected cost of search over these two angular extents are the same, both denoted as $g_{0}$.

As before, the cost of search for any point along the familiar route is assumed to be a monotonically increasing function of its distance from the start of search. Now, all points along $\overline{OO'}$ are further from $B'$ than any point along $\overline{AA'}$ to $B$. Therefore, the expected cost of searching for any set of points along $\overline{OO'}$ from $B'$, denoted $g_{OO'}$, must be greater than the expected cost of searching for any set of points along $\overline{AA'}$ from $B$, denoted $g_{AA'}$, i.e., $g_{OO'}>g_{AA'}$.

In order to compare the expected cost of search with respect to angular space, it is necessary to restrict the angular extent at $B$ which is used for the comparison, since $\gamma>\gamma'$. In other words, because $\angle OBA'>\angle OB'A'$ (or equivalently $\gamma>\gamma'$), a raw comparison of the average cost of search is unfair since the target (familiar route) subtends a smaller angle at the start of search, if searching begins at $B'$ rather than at $B$. To account for this difference, we consider only part of $\angle OBA'$ whose angular extent is equivalent to $\angle OB'A'$. The corresponding expected cost of search for the fraction of points along $\overline{AA'}$ which subtends angle $\gamma'$ at $B$ is denoted $g_{AA'}^{\gamma'}$. It is then straightforward to show that $g_{AA'}^{\gamma'}<g_{AA'}<g_{OO'}$. With angular adjustment, the expected cost of search from $B$ is thus

$$\left\langle g\right\rangle_{B}=\frac{\theta_{0}g_{0}+\gamma'g_{AA'}^{\gamma'}}{\theta_{0}+\gamma'}$$

and

$$\left\langle g\right\rangle_{B'}=\frac{\theta_{0}g_{0}+\gamma'g_{OO'}}{\theta_{0}+\gamma'}$$

Thus with adjustment for angular extent at the start of search,

$$\left\langle g\right\rangle_{B'}>\left\langle g\right\rangle_{B}$$

Hence the expected cost of search with respect to the angular extent of the familiar route is minimized when $\mu_{X}=x_{L}/2$ (or equivalently when $\Delta x=0$). This result is consistent with the analysis of search cost with respect to the linear spatial extent of the familiar route.
